# MRI of focal cortical dysplasia

**DOI:** 10.1007/s00234-021-02865-x

**Published:** 2021-11-27

**Authors:** Horst Urbach, Elias Kellner, Nico Kremers, Ingmar Blümcke, Theo Demerath

**Affiliations:** 1 Dept. of Neuroradiology, Medical Center – University of Freiburg, Breisacher Str. 64, 79106 Freiburg, Germany; 2 Dept. of Medical Physics, Medical Center – University of Freiburg, Freiburg, Germany; 3 Dept. of Neuropathology, University Hospital Erlangen, Friedrich-Alexander-University Erlangen-Nürnberg, Erlangen, Germany

**Keywords:** Focal cortical dysplasia, MRI, Lesions

## Abstract

Focal cortical dysplasia (FCD) are histopathologically categorized in ILAE type I to III. Mild malformations of cortical development (mMCD) including those with oligodendroglial hyperplasia (MOGHE) are to be integrated into this classification yet. Only FCD type II have distinctive MRI and molecular genetics alterations so far. Subtle FCD including FCD type II located in the depth of a sulcus are often overlooked requiring the use of dedicated sequences (MP2RAGE, FLAWS, EDGE) and/or voxel (VBM)- or surface-based (SBM) postprocessing. The added value of 7 Tesla MRI has to be proven yet.

Focal cortical dysplasia (FCD) are the most commonly resected epileptogenic lesions in children and the third most common lesions in adults [1]. They are defined as inborn (developmental), localized regions of malformed cerebral cortex, and encompass a broad spectrum of histopathological (light-microscopical) abnormalities [2]:

In a three-tiered light-microscopical classification proposed by the ILAE, FCD type I is a malformation with abnormal cortical layering, either compromising the radial (FCD type Ia) or the tangential composition of the 6-layered neocortex (FCD type Ib). The combination of both variants is classified as FCD type Ic [2]. Note, however, that very recently, the existence of FCD type Ib and Ic has been questioned [3].

FCD type II is a malformation with disrupted cortical lamination and specific cytological abnormalities, which differentiates FCD type IIa (dysmorphic neurons without balloon cells) from FCD type IIb (dysmorphic neurons and balloon cells) [4]. In 2011, Blumcke et al. added a FCD type III as a FCD type I in combination with hippocampal sclerosis (FCD type IIIa), with epilepsy-associated tumors (FCD type IIIb), adjacent to vascular malformations (FCD type IIIc), or in association with epileptogenic lesions acquired in early life (i.e., traumatic injury, ischemic injury, or encephalitis) (FCD type IIId) [2].

Note that the rare association between FCD type II with hippocampal sclerosis, tumors, or vascular malformations should not be regarded as FCD type III variant [2]. In addition to FCD defined as localized regions of malformed cerebral cortex so-called mild malformations of cortical development (mMCD) may cause epilepsy. In mMCD, the cortical architecture is intact, the cortex is absent of aberrant cells, but an excessive number of neurons in the molecular layer (type1) or the white matter (type 2) is found [4]. Mild malformation of cortical development with oligodendroglial hyperplasia (MOGHE) is another mild malformation of cortical development characterized by gray white matter blurring due to heterotopic neurons in the white matter and an increased number of normal-appearing oligodendroglial cells in the deep cortical and the juxtacortical white matter [5, 6].

Distinct molecular genetics alterations are so far confined to FCD type II; no consistent findings have been reported for mMCD or FCD I [7–9]. In FCD type II, mammalian target of rapamycin (mTOR) pathway mutations of genes within this pathway, including *AKT1*, *AKT3*, *DEPDC5*, *MTOR*, *NPRL2/3*, *PIK3CA*, *PIK3R2*, and *TSC1/2* mutations, can be found [10–12].

On MRI, FCD features are an increased cortical thickness (60–91% of FCD), a blurring of the gray/white matter junction (74–96% of FCD), a transmantle sign (75% of FCD type IIa, 94% of FCD type IIb), and/or an abnormal gyral/sulcal pattern [13–18]. mMCD (type 2) can be characterized by a signal increase of the white matter with blurring of the gray/white matter junction (Table 1). In young children (up to 3 years), MOGHE typically shows a T2- and FLAIR-hyperintense juxtacortical band (subtype I) (Fig. 3), which may represent hypomyelination. In older children, the band is not longer visible; instead, there is a reduced corticomedullary differentiation (subtype II) [6].

**Table 1:** ILAE classification of FCD, molecular genetics, and MRI findings

	Histology	Molecular genetics	MRI
FCD Ia	Radial microcolumns	None	Not directly visible, but may show blurring of the gray/ white matter junction due to heterotopic U-fiber neurons
FCD Ib	Tangential microcolumns		
FCD Ic	Radial and tangential microcolumns		
FCD IIa	Dysmorphic neurons	mTOR pathway mutations (*AKT1*, *AKT3*, *DEPDC5*, *MTOR*, *NPRL2/3*, *PIK3CA*, *PIK3R2*, *RHEB*, *TSC1/2*)	Increased cortical thickness, blurring of the gray/white matter junction, abnormal gyral/sulcal pattern
FCD IIb	Dysmorphic neurons + balloon cells		+ transmantle sign (94% of patients)
FCD IIIa	FCD I + hippocampal sclerois	None	Not directly visible, but may show white matter hypoplasia + white matter blurring
FCD IIIb	FCD I + epilepsy-associated tumors		?
FCD IIIc	FCD I + vascular malformation		?
FCD IIId	FCD I + early life event		?
mMCD 1	Ectopic neurons in molecular layer of neocortex	None	?
mMCD 2	Ectopic neurons in white matter		May show white matter blurring
MoGHE	Increased number of oligodendroglial cells + ectopic neurons in white matter	Mosaic *SLC35A2* variants	white matter blurring (in frontal lobe)

MRI abnormalities of FCD are often subtle and—as they usually do not change during life—often overlooked. The most overlooked lesion is a FCD in the depth of a sulcus (bottom of sulcus dysplasia) [19]. The transmantle sign—a funnel-shaped hyperintensity tapering towards the lateral ventricle—is only found in FCD type II [13, 17]. It is suggestive of a FCD type IIb, but not present in all FCD type IIb [10, 17]. The subtle abnormalities may be highlighted with specific MR sequences and postprocessing tools. In this review, we describe several strategies to enhance the visibility of FCD and correlate histopathological classifications and MRI findings.

## MRI protocols

MRI protocols to evaluate patients with drug-resistant focal epilepsies are largely standardized [20–22] (Table 2).

**Table 2:** Epilepsy-dedicated MRI protocol (3 T Magnetom Prisma, Siemens Healthcare, Erlangen, Germany)

MRI sequence	No. of slices/thickness (mm)	Voxel size (mm^3^)	TI/TR/TE/α (ms/ms/ms/°)	Acquisition time (min:s)
sag 3D MPRAGE	160/ 1	1 × 1 × 1	900/2000/2.26/12	4:40
sag 3D FLAIR-SPACE	160/1	1 × 1 × 1	1800/5000/388/var	6:52
ax 2D T2-TSE	42/3	0.4 × 0.4 × 3	5040/102/150	4:34
ax 2D T2*	23/5	0.7 × 0.7 × 5	639/ 19.9/20	2:33
cor 2D T2-STIR	40/2	0.4 × 0.4 × 2	100/5390/25/140	8:07
cor 2D FLAIR	68/2	0.7 × 0.7 × 2	2500/9000/87/150	4:14
ax 2D DWI-SE EPI	23/5	0.6 × 0.6 × 5	3400/85	0:46
sag 3D MP2RAGE	192/1	1 × 1 × 1	700, 5000/2000/2.9/4	8:52

*MPRAGE* magnetization prepared rapid gradient echo, FLAIR SPACE fluid-attenuated inversion recovery—sampling perfection with application-optimized contrasts by using flip angle evolution, *TSE* turbo spin echo, *STIR* short tau inversion recovery, *DWI* diffusion-weighted imaging, *SE* spin echo, *EPI* echo planar imaging, *TI* inversion time, *TR* repetition time, *TE* echo time, *α* flip angle, *var* variable flip angle.

## Key sequences

Within a MRI protocol at 3 Tesla, the 3D fluid-attenuated inversion recovery (FLAIR) sequence named SPACE, CUBE, VISTA, or depending on the MR vendor is the most relevant MRI sequence for the visualization of epileptogenic lesions [21, 22]. Nulling of the CSF signal helps to improve the visibility of hyperintense cortical lesions [22]. Although 2D FLAIR sequences have a higher in-plane resolution and signal to noise ratio (S/N), 3D FLAIR sequences with isotropic 1 mm^3^ voxel are preferred as they allow for multiplanar reformations, which may include reformations along and perpendicular to the orientation of the FCD [20, 22, 23].

The magnetization‐prepared rapid gradient‐echo (MPRAGE) sequence and equivalent 3D spoiled gradient echo and 3D turbo field echo sequences with isotropic millimetric voxel resolution (e.g., 1 × 1 × 1 mm^3^) are other key sequences. These sequences allow not only for optimal evaluation of brain anatomy and morphology but are also used for voxel-based analysis including volumetry and postprocessing [24–31].

**Fig. 1:**
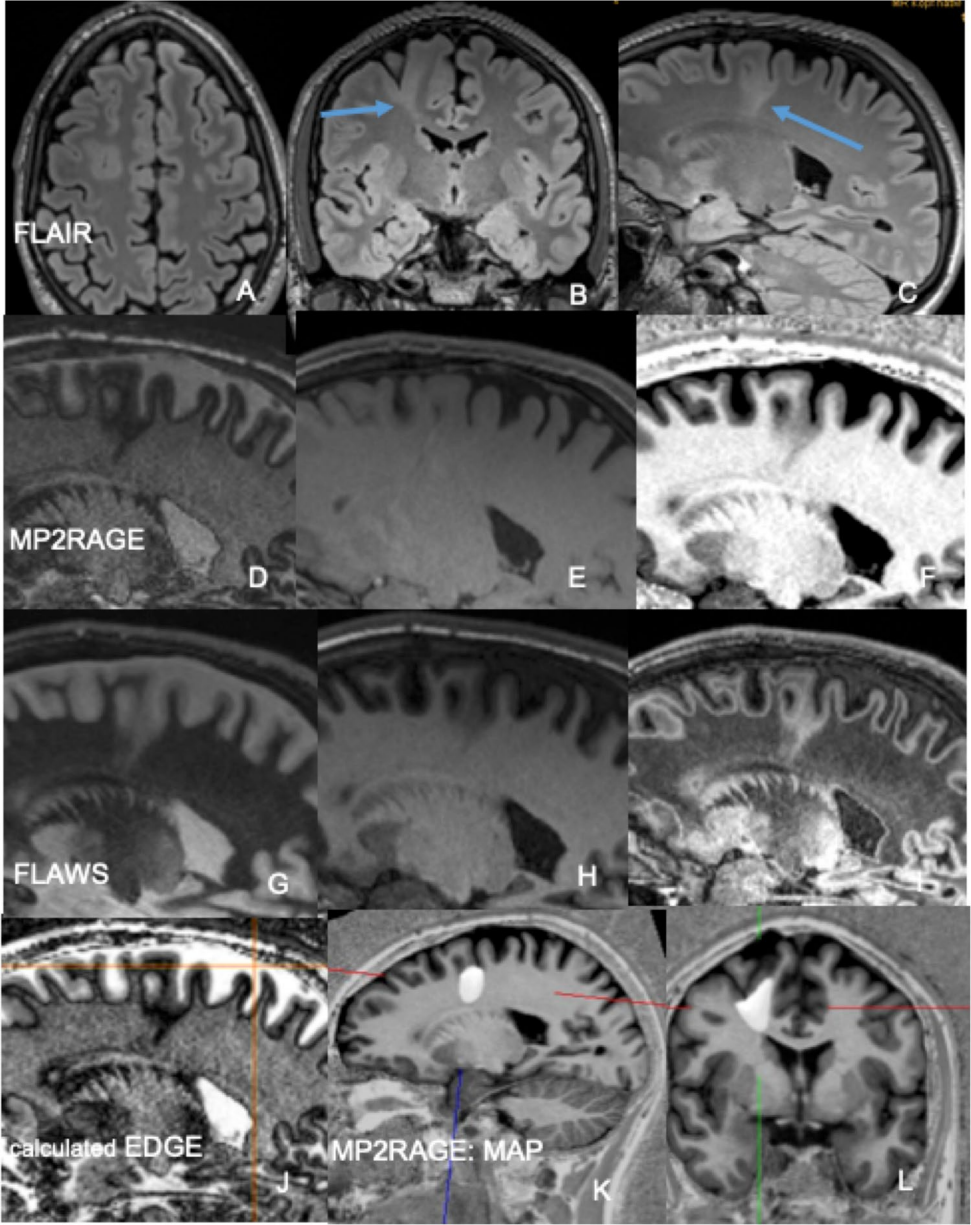
FCD type IIb in the depth of the right superior frontal sulcus. **A**–**C** 3 Tesla axial, coronal, and sagittal 3D FLAIR SPACE images show a thickenend cortex and a hyperintense transmantle sign tapering towards the frontal horn of lateral ventricle (**B**, **C**: arrow). **D**–**F** 3 Tesla sagittal MP2RAGE images at inversion times TI of 700 ms (**D**) and 2500 ms (**E**). Calculated so-called unified image (**F**). **G**–**I** 3 Tesla sagittal FLAWS images at inversion times TI of 409 ms (**G**) and 1160 ms (**H**). Calculated minimum intensity image (**I**). **J** 3 Tesla calculated sagittal EDGE image at an inversion time of 442 ms according to Bydder and Young (1995) and Hornak (2008). **K**–**L** MAP-postprocessed MP2RAGE images after inverse normalization and co-registration of the CNN output map to the unified images

The MP2RAGE sequence is a MPRAGE sequence with two inversion pulses at 700 ms and 2500 ms, respectively. From the two images, a so-called unified image is calculated using the formula $\mathrm{MP}2\mathrm{RAGE}=\frac{\mathrm{contrast TI}1 \times \mathrm{ contrast TI}2}{{\mathrm{contrast T}1}^{2} + {\mathrm{contrast TI}2}^{2}}$ (Fig. 1).

The MP2RAGE sequence produces images with a higher B_1_ homogeneity than the MPRAGE sequence and is therefore particularly suited for postprocessing [31–35]. The higher B_1_ homogeneity is also the reason that 7 Tesla scanners are routinely equipped with this sequence [36–40].

The fluid and white-matter suppression (FLAWS) sequence is similar to the FLAIR sequence; however, not only the CSF but also the white matter signal is nulled. Two 3D sets with isotropic 1 mm^3^ voxel are acquired in an interleaved acquisition scheme with two different inversion times (TI): TI_1_ suppresses the white matter signal and TI_2_ suppresses the CSF signal. From both data sets, a set of synthetic minimum FLAWS contrast images is calculated which can be regarded as a gray matter specific image [41, 42] (Fig. 1).

The 3D Edge-Enhancing Gradient Echo sequence is a MPRAGE sequence with an inversion time of 442 ms [43]. At this inversion time, gray and white matter have equal signals but opposite phases and voxels with a mixture of gray and white matter (e.g., at the gray-white boundary) will have cancelation of longitudinal magnetization producing a thin area of signal void at the normal boundary (Fig. 1). Contrast to noise ratio (C/N) is reported to be higher than on MPRAGE and FLAIR sequences [43].

## Quantitative MRI sequences

Quantitative MRI sequences measure tissue parameters such as the T1-, T2-, T2* relaxation times, or the proton density (PD) free from hardware-effects. However, they require the acquisition of several sequences including gradient echo sequences to correct for inhomogeneities of B_0_ and B_1_, and for insufficient spoiling of the transverse magnetization [44]. For T1and PD mapping, the variable flip angle method acquiring two spoiled gradient echo data sets at different flip angles is used [45]. A strategy for voxel-wise measurement of the T2 relaxation time is to acquire four fast spin echo datasets with different echo times (TEs), e.g., TE = 13, 67, 93, and 106 ms. Mapping of both T2* and B0 inhomogeneities (to correct T1 data) is based on the acquisition of eight multiple-echo gradient echo (GE) datasets, e.g., 10, 16, 22, 28, 34, 40, 46, and 52 ms [46, 47]. Quantitative MRI sequences can be used to calculate synthetic magnetization-prepared rapid gradient-echo (MPRAGE) sequences which are—due to their high B_1_ field homogeneity—particularly suited for postprocessing.

3D MR fingerprinting (MRF) is another quantitative MRI technique. Acquired signals from a single ≈ 12 min 3D sequence with variable combinations of hundreds to thousands of TRs and flip angles are compared with those in a dictionary that contains signal evolutions from a wide range of physiologically relevant combinations of T1 and T2 [49). The acquired signal from each voxel is then assigned to the entry in the dictionary that best matches the signal evolution [47]. 3D MRF was reported to show additional information in 4 of 15 patients with FCD but is not in clinical routine yet [48].

## Postprocessing

MRI features such as the cortical thickness, the gyral/sulcal pattern, or blurring of the gray/white matter junction can be computed semiautomatically using voxel-based (VBM) or surface-based morphometry (SBM). Texture analysis computing cubic volume sampling around each voxel to calculate second- and third-order textural features has also been described [49].

In a standard setting, T1-weighted data sets with 1 × 1 × 1 mm^3^ are converted from DICOM to NIfTI format, undergo intensity non-uniformity correction, and are warped to a common template such as the MNI152 template. Other sequences, e.g., FLAIR, can be linearly mapped to the T1-weighted data sets. Next, gray matter (GM), white matter (WM), and CSF compartments are segmented.

**Fig. 2:**
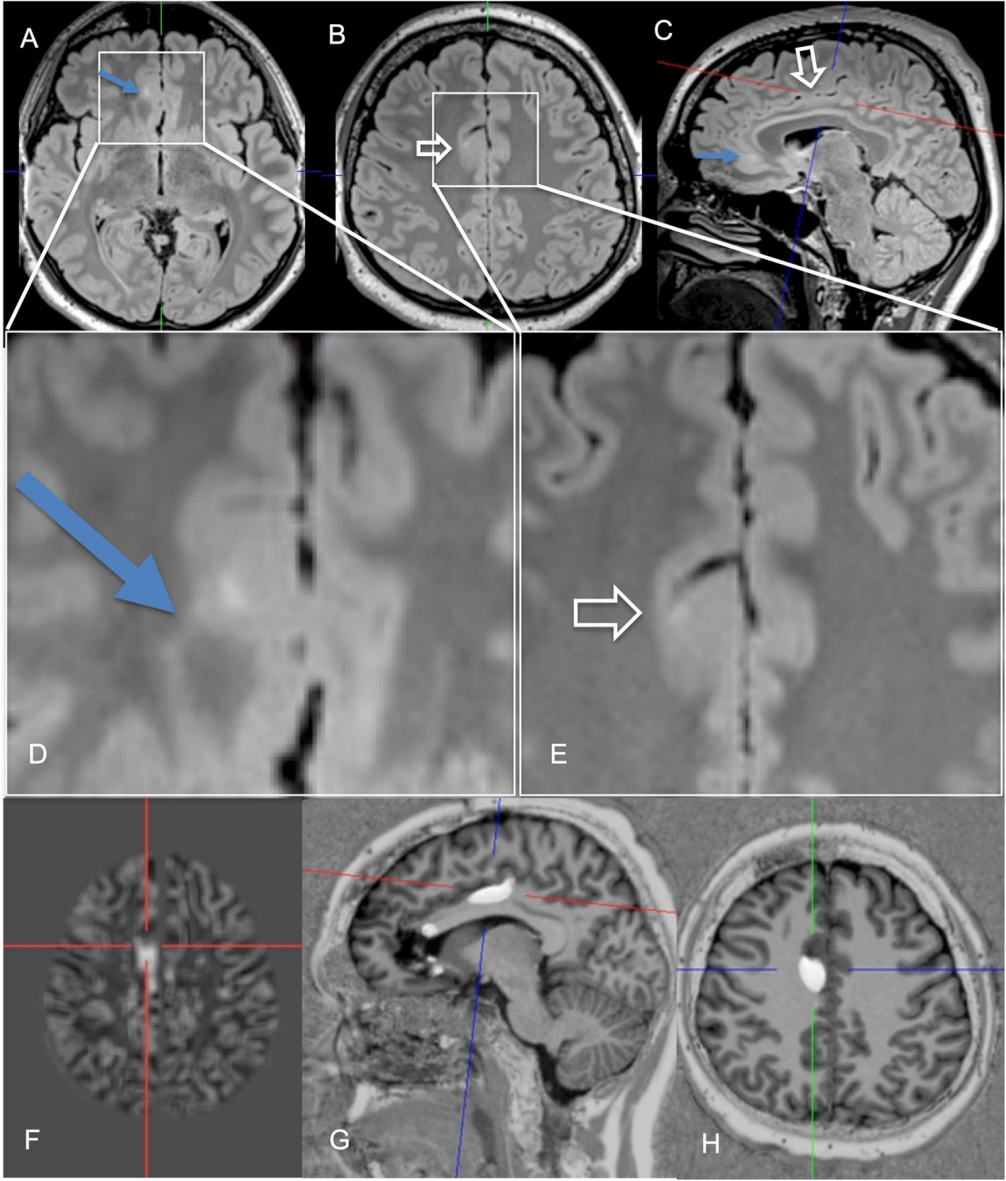
A 24-year-old man with two FCD in the right cingulate gyrus. The anterior one with a transmantle sign was visually detected on a 3 Tesla 3D FLAIR sequence with isotropic 1 mm^3^ voxels (**A**, **C**–**D**: arrow). The posterior one was detected with the aid of the morphometric analysis program only (**B**, **C**, **E** hollow arrow, **F** crosshair on so-called junction image before first surgery, **G**–**H** crosshair on co-registered MP2RAGE and probability maps after second surgery). While the anterior lesion was classified as FCD IIb, no FCD was diagnosed for the posterior lesion. As the patient did not get seizure-free the posterior lesionectony was extended and histology now revealed a FCD IIa

For VBM, several feature maps (e.g., thickness, extension, junction maps in the MAP tool) are computed voxel-wise [24]. For SBM, the gray/white matter boundary is tessellated and the folded surface tessellation inflated [28]. It allows for measuring the cortical thickness from its vertices and to calculate features such as intensity gradients within the cortex itself, but also features such as the gyral curvature, sulcal depth, or local cortical deformation [29, 50]. Most VBM and SBM algorithms however focus on the gray/white matter transition zone as the most prevalent feature of FCD. They compute features such as a smooth transition from the gray to the white matter. Features are typically analyzed with respect to a nominal distribution (*z* scores) requiring the use of data bases of healthy controls [24, 28, 51]. Machine learning tools with convolutional neural networks (CNN) are increasingly incorporated in VBM and SBM tools and trained with manually labeled ground-truth data to find FCD approaching accuracies of close to 90% so far [25, 28, 31, 35, 52] (Figs. 1–2) (Table 3).

**Table 3:** Overview of the results of various VBM and SBM tools

Study	Sequence	Method	Sensitivity, specificity
Hong et al. 2014	MPRAGE	SBM + linear discriminant analysis	0.74, 1.00
Hong et al. 2017	MPRAGE, 3D FLAIR, DTI	SBM + linear discriminant analysis	n.a
Jin et al. 2018	MPRAGE	SBM + CNN	0.74, 0.90 AUC 0.75
Wang et al. 2015	MPRAGE	VBM: MAP	0.9, 0.67
David et al. 2021	MPRAGE	VBM: MAP + CNN	0.81, 0.84
Sun et al. 2021	MPRAGE	VBM: MAP	0.43, 0.87
Gill et al. 2021	MPRAGE + 3D FLAIR	SBM + CNN	0.87, 0.89
Demerath et al. 2021	MP2RAGE	VBM: MAP + CNN	0.82, 0.34

## Discussion

Fifteen to 30% of patients with drug-resistant epilepsy are considered to be MRI negative; that is, no structural lesion is identified [53, 54]. However, MRI-negative is a misnomer. It comprises patients without a MRI lesion and those, in which a subtle MRI lesion, which however has a histopathological substrate, overlooked. Most overlooked lesion are FCD type 2; the rate is higher for FCD type IIa than type IIb [55]. The rate of overlooked lesions is likely around 30% [28, 55–57], but may be as high as 41% or even 78% [25, 26, 58].

There is strong agreement that the visibility on MRI depends on the FCD type: FCD type II are visible on MRI provided adequate image quality on 3 Tesla machines and postprocessing is achieved. It has been reported that 7 Tesla is superior to 3 Tesla; however, post-processed 7 Tesla MP2RAGE images have been compared with 3 Tesla MPRAGE images [37, 38]. This comparison is biased as postprocessing using MP2RAGE images already displays FCD with larger volumes and higher *z* scores [34].

**Fig. 3:**
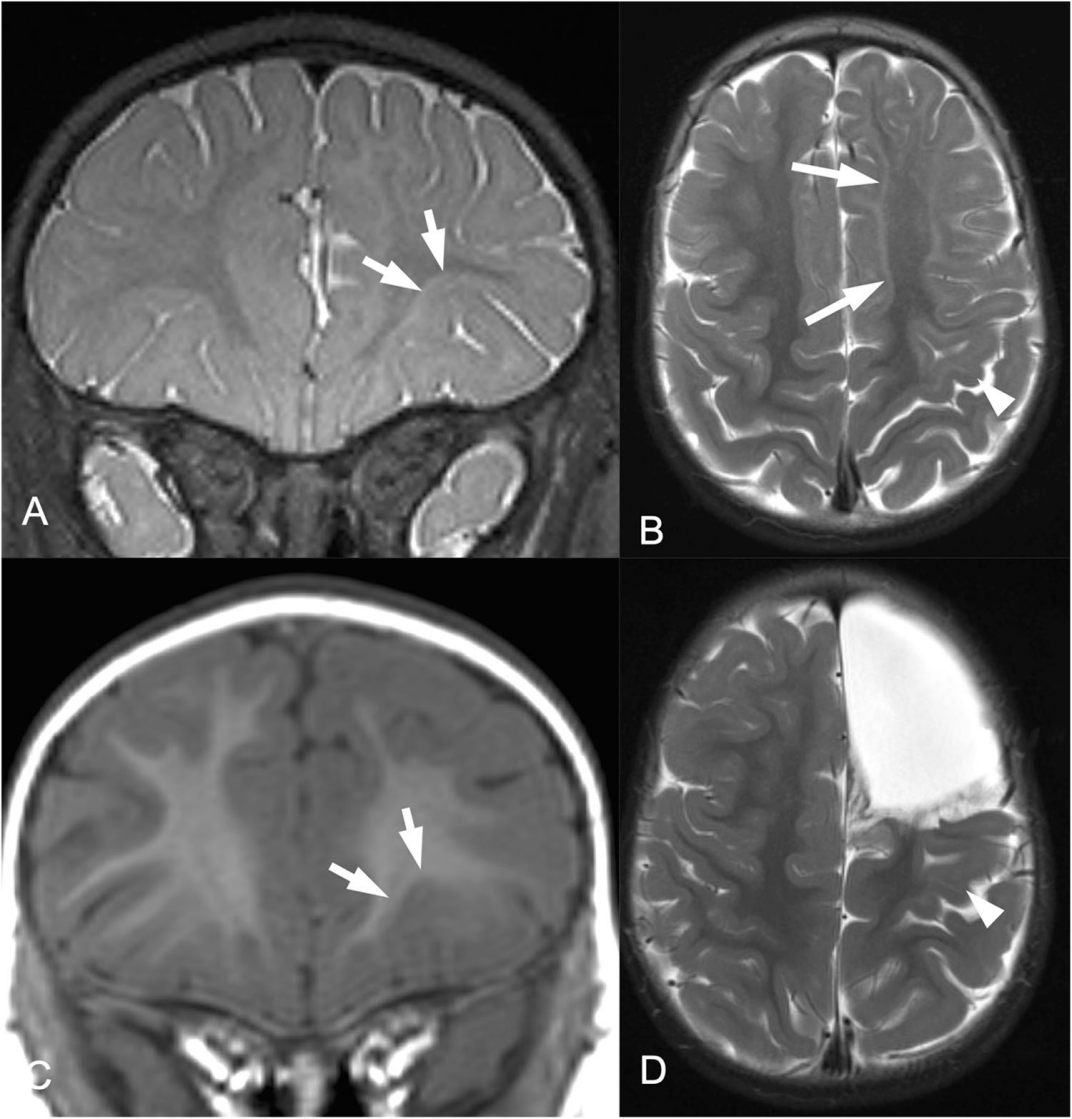
Mild malformation of cortical development with oligodendroglial hyperplasia (MOGHE) in the left frontal lobe in a 3-year-old girl. **A**–**C** 3 Tesla coronal 3D T2 SPACE (**A**), axial T2 TSE (**B**), coronal T1 MPRAGE (**C**), and axial T2 TSE (**D**) images. T2-weighted images show a juxtacortical hyperintense signal band (**A**–**B**, arrows), extending into the left precentral gyrus (**B**, arrowhead). On the MPRAGE image, the T2-hyperintense band looks like a subtle blurring of the gray-white matter junction (**C** arrows). **D** Axial T2w image after subtotal left frontal lobe resection. Despite residual lesion in the left precentral gyrus, the patient was seizure-free (Engel IA) after 3 months

There is limited agreement with respect to the visibility of FCD type I and III [22]. The abnormal arrangement of cortical neurons in FCD I and FCD III should not be visible on MRI as the cellular density is not changed [3]. However, the U-fiber layer beneath contains an excessive number of heterotopic neurons leading to a blurring of the gray/white matter junction. These displaced neurons form complex synaptic plexus within the U-fiber layer; some axons of which ascend into the cortex to be integrated into synaptic networks [59]. Mild malformation of cortical development with excessive white matter neurons (mMCD type 2) and with oligodendrogial hyperplasia (MOGHE) (Fig. 3) also produce a blurring of the gray/white matter junction on T2-weighted/ FLAIR sequences.

The difficult diagnosis of mMCD and FCD type I is also indicated by the low intra-rater agreement documented in a blinded classification of 26 specimens by eight neuropathologists. A significant agreement was reached for FCD type II only [60]. Furthermore, the microcolumnar organization of FCD type Ia resembles neuronal radial migration streams during corticogenesis [61, 62] and may result, therefore, from delayed or arrested maturation at mid-gestation [10]. This also holds true for the temporal pole abnormalities associated with hippocampal sclerosis which may be FCD type IIIa but also may show a reduced number of axons on diffusion-mesocopic imaging [63]. The reduced number of axons rather indicates a maturation disorder and not a FCD.

Correlation of histology and MRI is further impeded by a significant number of patients with MRI-visible FCD who get seizure-free following surgery but show unremarkable histopathology (so-called MRI-positive, histology-negative FCD) (Fig. 2). In these patients, a neuropathological sampling error is the most likely explanation for missing the diagnosis.

Postprocessing has dramatically improved the detection rate of FCD. Thus, it is recommended to use a postprocessing tool for every patient with a drug-resistant focal epilepsy. Among the different tools, the VBM tool morphometric analysis program (MAP) is the most widely used tool; it has been integrated in standard presurgical workflows of over 60 epilepsy centers in 22 different countries [35, 55, 64]. The MAP tool has independently been validated for its clinical benefits against expert neuroradiological assessments [26, 34, 54,] with potential impact on further, also invasive presurgical patient management [34, 57]. Surface-based (SBM) tools at least theoretically allow for a better analysis of the cortical layers itself; however, the superiority to VBM has not been shown yet [20–30, 51, 65]. Multiple stand-alone tools also processing multiple including quantitative sequences are currently being developed [25, 27, 28, 30, 31, 47, 52, 64, 66]. Which postprocessing tool will prevail is at the end also a matter of availability and ease of use [64].

Due to their higher S/N, which is theoretically proportional to the magnetic field strength B_0_, more FCD should be detectable on 7 Tesla compared to 3 Tesla scanners. In practice, however, stronger B_1_ field inhomogeneities at 7 Tesla impede the detection of subtle signal differences especially at the gray/white matter junction. The higher susceptibility (*χ* ≈ B_0_) improves the visibility of lesions with paramagnetic substances but also comes with more artifacts at brain interfaces to air-filled bony structures. Wang et al. published the largest comparative study so far, a prospective cohort of previously MRI-negative classified 67 patients [37]. They investigated the additional value of 7 T MAP using the MP2RAGE sequence and detected 25% (6 of 24 patients) more lesions compared to 3 T MAP based on the MPRAGE sequence. As MP2RAGE images show a higher contrast and a higher contrast-to-noise ratio than MPRAGE images even at the same field strength, the meaningfulness is limited [34].

Current recommendations stress the technical challenges (use of dielectric pads to limit B_1_ field inhomogeneities, patient’s discomfort including dizziness, longer scanning times, larger flip angle variations in 3D SPACE sequences, etc.) and suggest the use of 7 Tesla MRI to be confined to 3 Tesla negative cases [36, 39, 40, 67].

## Conclusion

Albeit, there are standardized MRI protocols; FCD detection likely benefits from “newer” dedicated sequences (MP2RAGE, FLAWS, EDGE) and voxel- or surface-based postprocessing including the comparison with a data base of healthy controls. Only FCD type II have clear histopathological and MRI characteristics.

## Data Availability

On reasonable request to corresponding author, the underlying data can be accessed.
